# Complete Genome Sequence of the Extremely Halophilic Archaeon *Haloarcula hispanica* Strain N601

**DOI:** 10.1128/genomeA.00178-14

**Published:** 2014-03-13

**Authors:** Jiun-Yan Ding, Pei-Wen Chiang, Mei-Jhu Hong, Mike Dyall-Smith, Sen-Lin Tang

**Affiliations:** a Biodiversity Research Center, Academia Sinica, Taipei, Taiwan; b School of Biomedical Sciences, Charles Sturt University, Wagga Wagga, Australia

## Abstract

*Haloarcula hispanica* has been widely used in haloarchaeal studies, particularly in the isolation of haloviruses. The genome of strain N601, a laboratory derivative of the type strain ATCC 33960, was sequenced. Several potentially significant differences from the published sequence of the type strain (CGMCC 1.2049 = ATCC 33960) were observed.

## GENOME ANNOUNCEMENT

*Haloarcula hispanica* is an extremely halophilic archaeon, originally isolated from a solar saltern in Spain (1). Being easy to cultivate and genetically manipulate, this species is an attractive model archaeon and has been used in many studies (2–9), including the isolation of novel haloviruses (10–13). The genome sequence of strain ATCC 33960, the type strain of *H. hispanica*, has been reported (14) and consists of two main chromosomes and one megaplasmid. Here we report the genome sequence of *H. hispanica* strain N601, a laboratory-derived mutant that arose during routine cultivation of strain ATCC 33960^T^.

*Haloarcula hispanica* N601 was cultivated in 23% magnesium minimal medium (MGM) (15). Optical maps were prepared at Yourgene Bioscience, Inc. (New Taipei, Taiwan), and used to orientate contig sequences. DNA was extracted using the cetyltrimethylammonium bromide (CTAB) method (16) and sequenced at Mission Biotech, Inc. (Taipei, Taiwan), using a Roche GS FLX Titanium system. Using Newbler version 2.0.01.14 (Roche), we assembled 26 contigs from the pyrosequencing reads, and the optical maps allowed 14 of these contigs to be aligned with the major chromosome and 1 contig with the large plasmid. To sequence the second chromosome and the smaller plasmid, these replicons were separated by pulsed-field gel electrophoresis, the DNA was recovered by electroelution (17), and sequencing was performed using both the Roche GS Junior platform and the Illumina genome analyzer II platform. Illumina reads were mapped to the major chromosome and large plasmid sequences. The remaining reads were assembled together with unused 454 contigs using the CLC Genomics Workbench 6 (CLC Bio) software. rRNA operons and regions that differed from the previously published sequence were PCR amplified with Phusion high-fidelity DNA polymerase (Thermo Scientific) and cloned and Sanger sequenced at Genomics BioSci & Tech, Inc. (New Taipei, Taiwan).

The *H. hispanica* N601 genome is 3,901,495 bp in length, consisting of two chromosomes (chrI, 3,006,708 bp, and chrII, 363,208 bp) and two plasmids (pHH406, 405,815 bp, and pHH126, 125,764 bp). The overall GC content is 62.43%. The genome contains 3,932 putative protein-coding genes, of which 2,605 were assigned to clusters of orthologous groups (COG) families and 658 were mapped to KEGG pathways (18). The coding density was 87.13%. There are 48 tRNA genes, 3 rRNA operons, 2 noncoding RNAs, and 1 clustered regularly interspaced short palindromic repeat (CRISPR) array. Major differences detected between the sequence of strain N601 and that of strain ATCC 33960^T^ (CGMCC 1.2049) include (i) the presence of 4 replicons (compared to 3), (ii) a prophage-like region in chrI (base position, 22,557 to 34,957) not seen in ATCC 33960^T^, (iii) a region containing two hypothetical protein-coding genes in chrI (base position, 712,834 to 714,267) not seen in ATCC 33960^T^, and (iv) a truncation event in chrI of strain N601 (loci HISP_06336 and HISP_06337). The genome sequence of strain N601, and particularly the differences from strain ATCC 33960^T^, may be useful in understanding the physiology, genome dynamics, and halovirus-host interactions within this species.

### Nucleotide sequence accession numbers.

The genome sequences of *H. hispanica* N601 and its annotation have been deposited at GenBank under the accession numbers CP006884 (chrI), CP006885 (chrII), CP006886 (pHH126), and CP006887 (pHH406).
